# Structural insights into the enzymatic activity and potential substrate promiscuity of human 3-phosphoglycerate dehydrogenase (PHGDH)

**DOI:** 10.18632/oncotarget.22327

**Published:** 2017-11-06

**Authors:** Judith E. Unterlass, Robert J. Wood, Arnaud Baslé, Julie Tucker, Céline Cano, Martin M.E. Noble, Nicola J. Curtin

**Affiliations:** ^1^ Northern Institute for Cancer Research, Medical School, Newcastle University, Newcastle upon Tyne, UK; ^2^ Cancer Research Technology, Discovery Laboratories, Babraham Research Campus, Cambridge, UK; ^3^ Institute for Cell and Molecular Biosciences, Newcastle University, Newcastle upon Tyne, UK; ^4^ Northern Institute for Cancer Research, School of Chemistry, Newcastle University, Newcastle upon Tyne, UK; ^5^ Present address: Science for Life Laboratory, Department of Medical Biochemistry and Biophysics, Karolinska Institutet, Stockholm, Sweden

**Keywords:** 3-phosphoglycerate dehydrogenase, substrate and cofactor specificity, serine synthesis

## Abstract

Cancer cells reprogram their metabolism and energy production to sustain increased growth, enable metastasis and overcome resistance to cancer treatments. Although primary roles for many metabolic proteins have been identified, some are promiscuous in regards to the reaction they catalyze. To efficiently target these enzymes, a good understanding of their enzymatic function and structure, as well as knowledge regarding any substrate or catalytic promiscuity is required. Here we focus on the characterization of human 3-phosphoglycerate dehydrogenase (PHGDH). PHGDH catalyzes the NAD^+^-dependent conversion of 3-phosphoglycerate to phosphohydroxypyruvate, which is the first step in the *de novo* synthesis pathway of serine, a critical amino acid for protein and nucleic acid biosynthesis. We have investigated substrate analogues to assess whether PHGDH might possess other enzymatic roles that could explain its occasional over-expression in cancer, as well as to help with the design of specific inhibitors. We also report the crystal structure of the catalytic subunit of human PHGDH, a dimer, solved with bound cofactor in one monomer and both cofactor and *L*-tartrate in the second monomer. *In vitro* enzyme activity measurements show that the catalytic subunit of PHGDH is still active and that PHGDH activity could be significantly inhibited with adenosine 5’-diphosphoribose.

## INTRODUCTION

**Figure 1:**
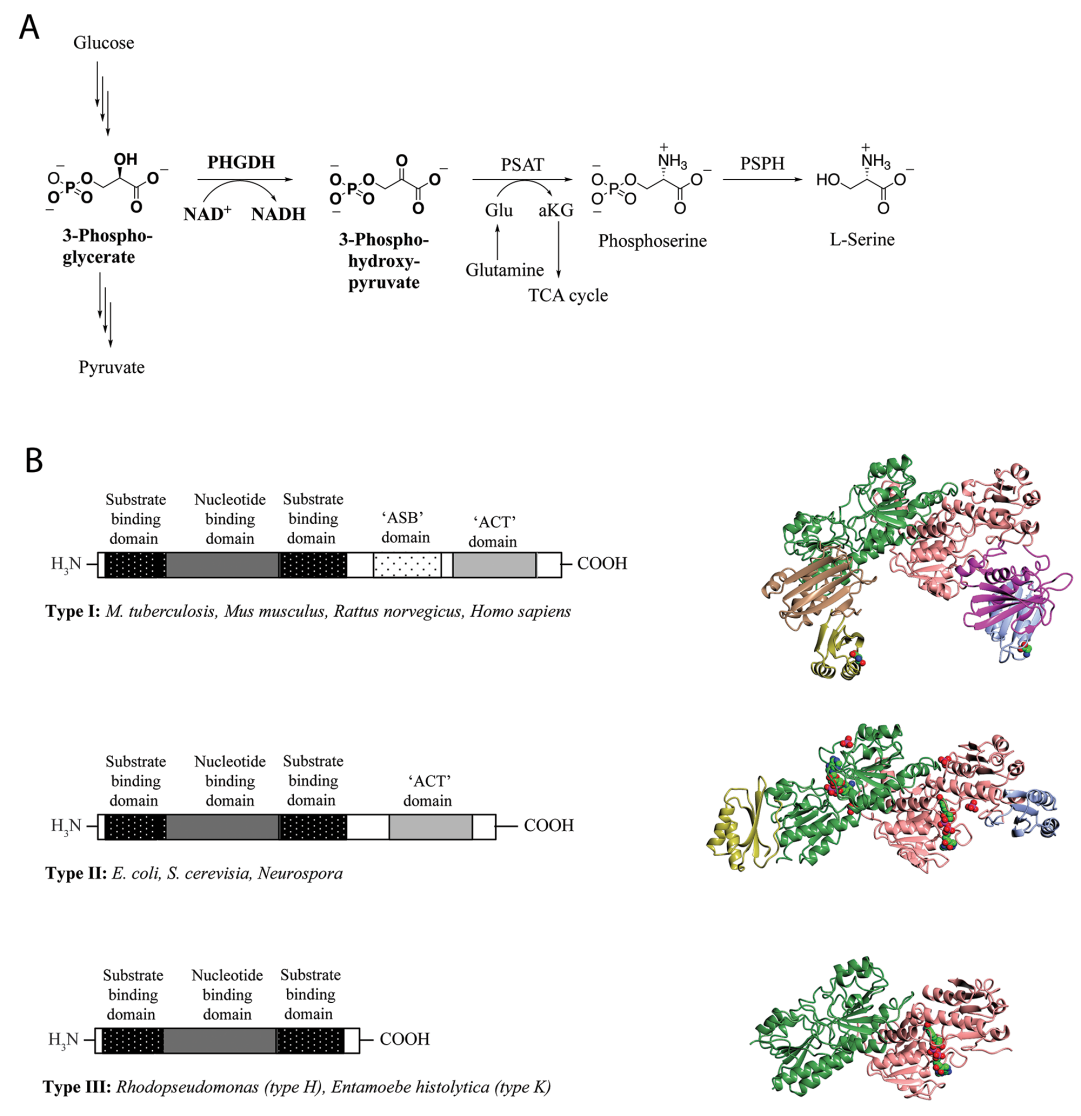
*L*-Serine synthesis pathway and basic domain structure of PHGDH **(A)** 3-Step synthesis scheme of endogenous L-serine starts with the oxidation of 3-phosphoglycerate to 3-phosphohydroxypyruvate by PHGDH and simultaneous reduction of the cofactor NAD^+^ to NADH. The subsequent transamination reaction is catalyzed by phosphoserine aminotransferase (PSAT), which uses glutamate (Glu) as nitrogen donor and thereby produces phosphoserine and α-ketoglutarate (αKG). Dephosphorylation of phosphoserine by phosphoserine phosphatase (PSPH) gives rise to L-serine. **(B)** Basic domain structure found within the three enzyme types of PHGDH shaded by domain. Additional amino acids at the N-terminus are not explicitly shown as variations in length and composition of this part of the protein depend on the species. Two forms of the type III enzyme exist depending on whether lysine (type K) or histidine (type H) is present at the active site [8] (left). Crystal structures of representative family members of the different types of PHGDH. For a better comparison, all enzymes are shown as dimers, although active PHGDH from *M. tuberculosis* and *E. coli* form a tetramer. The substrate- and nucleotide-binding domain is shown in green/rose, the ASB domain is shown in magenta/brown and the ACT domain is shown in blue/yellow. If present in the crystal, the cofactor NAD+ is depicted in spheres and colored by atom type (carbon in green) (right).

3-Phosphoglycerate dehydrogenase (PHGDH) catalyzes the first step in the *de novo* serine synthesis pathway, *i.e.* the NAD^+^-dependent conversion of 3-phosphoglycerate (3-PG) to phosphohydroxypyruvate (PHP). PHGDH diverts the glycolytic flux towards producing serine, which in turn is metabolized and incorporated into a variety of biomolecules (Figure 1A) including glycine, thereby providing a major source of one-carbon units for the synthesis of purine and pyrimidine nucleotides.

Normal cells metabolize glucose via glycolysis to pyruvate, which then can be further oxidized in the tricarboxylic acid cycle to carbon dioxide and water with concomitant synthesis of ATP. Interestingly, cancer cells mainly metabolize glucose through fermentation even in the presence of saturating amounts of oxygen, resulting in increased lactate production and a reduced ATP yield [1]. This upregulation of aerobic glycolysis – termed “Warburg effect” after its discoverer Otto Warburg - results in less energy in the form of ATP from each glucose molecule. However, it also allows for the diversion of the glycolytic flux into biomass generation, which is of particular importance for highly proliferating cells such as cancer cells. The Warburg effect is an exploitable difference between normal and cancer cells and provides new avenues for targeting cancer. Against this background, PHGDH, as a major enzyme in the diversion of glycolytic flux towards serine synthesis, is of particular interest.

Recent work highlighted the importance of human PHGDH in certain cancer types with amplified PHGDH, *e.g.* breast cancer and melanoma, with PHGDH knockdown resulting in reduced cancer cell growth [2-4, 10]. This sensitivity indicates a potential therapeutic use for PHGDH inhibitors in tumors expressing high levels of the target enzyme. In this context, recently developed PHGDH inhibitors have shown promising results in the setting of cells with high PHGDH expression/ *PHGDH* amplification [5, 6].

PHGDH is ubiquitously expressed in all organisms, and exists in at least three different basic structural forms, referred to as type I, II and III (Figure 1B) [7]. These forms do not appear to be strictly life-domain specific as mammalian PHGDH shows structural homology with the enzyme from the pathogenic bacterium *Mycobacterium tuberculosis*. Both the human and the mycobacterial enzymes belong to the structurally most complex type, type I. All three types of PHGDH contain two common domains: the substrate-binding domain and the cofactor-binding domain. Type I enzymes contain two additional regulatory domains, the ACT (aspartate kinase-chorismate mutase-tyrA prephenate dehydrogenase) and ASB (allosteric substrate binding) domains. For certain species, the ACT domain has been reported to function as a binding site for serine to provide feedback inhibition, although this regulatory mechanism could not be confirmed for human PHGDH [8].

To date, no full-length crystal structure of any mammalian PHGDH is known, although a structure has been solved for the closely related *M. tuberculosis* PHGDH (PDB 1YGY) [9]. PHGDH from *M. tuberculosis* crystallizes as a tetramer in which the catalytic domains adopt the same conformation in all four subunits, whereas the regulatory domains adopt differing conformations. For human PHGDH, a structure of the core domain comprising the cofactor-binding site (amino acids (aa) 93-298) has been elaborated as a tool for a fragment-based inhibitor design [10]. A structure of the complete catalytic subunit of human PHGDH (sPHGDH, aa 3-314) (PDB 2G76, http://www.thesgc.org/structures/2g76#mandm) has also been deposited. Unlike *M. tuberculosis* PHGDH, human sPHGDH formed a dimer rather than a tetramer in the crystal, probably due to the truncated protein lacking the regulatory domains. sPHGDH was crystallized in the presence of 0.1 M malate, and *D*-malate was observed bound in the active site. Malate, an analogue of the substrate 3-PG, was shown to be favorably aligned for the catalytic reaction, including the formation of a salt bridge between the carboxylic group of malate and Arg235 of the enzyme [8]. Thus, PHGDH might catalyze a reaction with malate under certain environmental conditions. Recently, human PHGDH was also shown to be able to reduce α-ketoglutarate, a structural analogue of PHP, to 2-hydroxyglutarate (2-HG), both *in vitro* and in the *PHGDH*-amplified breast cancer cell line MDA-MB-468 [11].

These findings strongly suggest that PHGDH shows a substrate and catalytic promiscuity, and alternative substrates or reactions might become relevant under certain biological conditions. This promiscuity of PHGDH could be particularly relevant in explaining the role of PHGDH in cancer and may suggest PHGDH as a tractable target for inhibitor design. Although the main role identified for PHGDH so far is in contributing to serine synthesis, this might not explain why certain cancers seem to rely on PHGDH, as serine is also taken up exogenously. The existence of an additional function is further suggested by the observation that PHGDH-depletion in PHGDH-amplified cells results in decreased cell proliferation that cannot be rescued through excessive addition of serine in the cell media [2, 4].

Here, in order to better understand the substrate and catalytic promiscuity of human PHGDH and to identify other potential roles of PHGDH that might be relevant in diseases, such as cancer, we have developed and applied functional and binding assays for PHGDH, and have solved structures for the catalytic domain in complex with its cofactor NAD^+^ both alone and together with the substrate analogue *L*-tartrate. With these tools, we have investigated the properties of substrate and co-factor analogues that are required to permit binding to sPHGDH, revealing a remarkably permissive active site that might be exploited in drug discovery.

## RESULTS

### Enzyme activity assay for PHGDH

**Figure 2:**
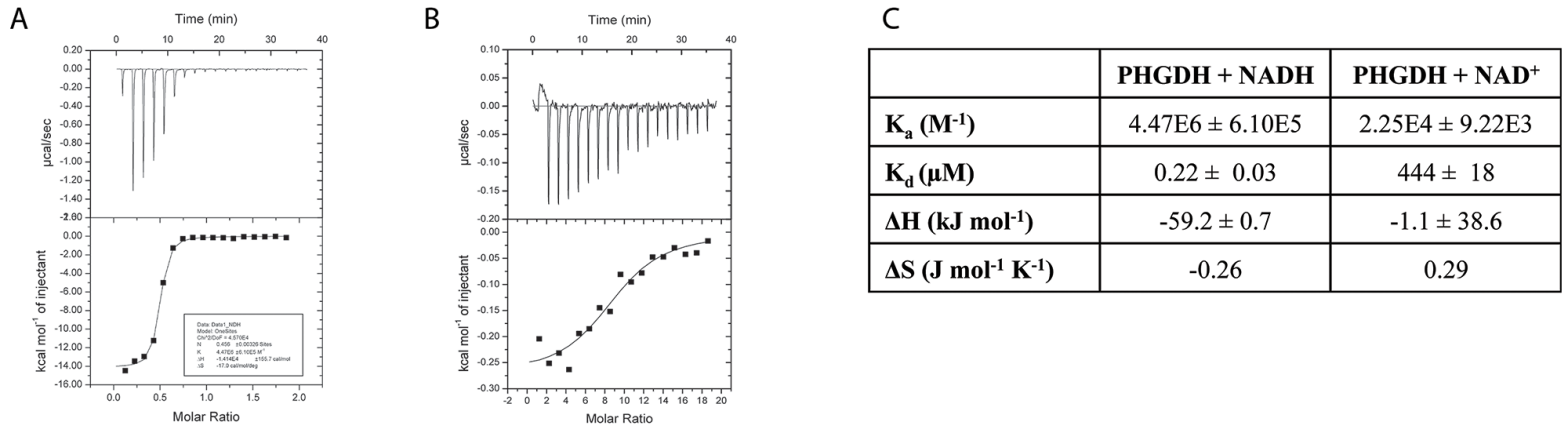
Binding of NAD^+^ and NADH to PHGDH as investigated by ITC **(A)** 0.5 mM NADH or **(B)** 5 mM NAD^+^ was titrated into 0.05 mM PHGDH in 25 mM HEPES, pH 7.5, 100 mM NaCl and 0.5 mM TCEP. Top panel: raw data for 1 x 0.5 μL, followed by 17 x 2 μL injections of NADH or NAD^+^ into the isothermal cell containing 0.05 mM PHGDH. Data were corrected for heat of cofactor dilution by subtracting the heats from cofactor to buffer titration. Bottom panel: integrated heats from the peaks in the top panel plotted against the molar ratio of cofactor to PHGDH. The line of best fit to the data was plotted as obtained by non-linear regression using the built-in one-site fit model of the ORIGIN software. **(C)** Data obtained from the one-site fit model in ORIGIN. Stoichiometry of the reaction (= number of binding sites, N), changes in entropy (ΔS) and enthalpy (ΔH) as well as the association constant (K_a_) were determined. Binding constant (K_D_) was calculated by taking the reciprocal of the K_a_.

**Figure 3:**
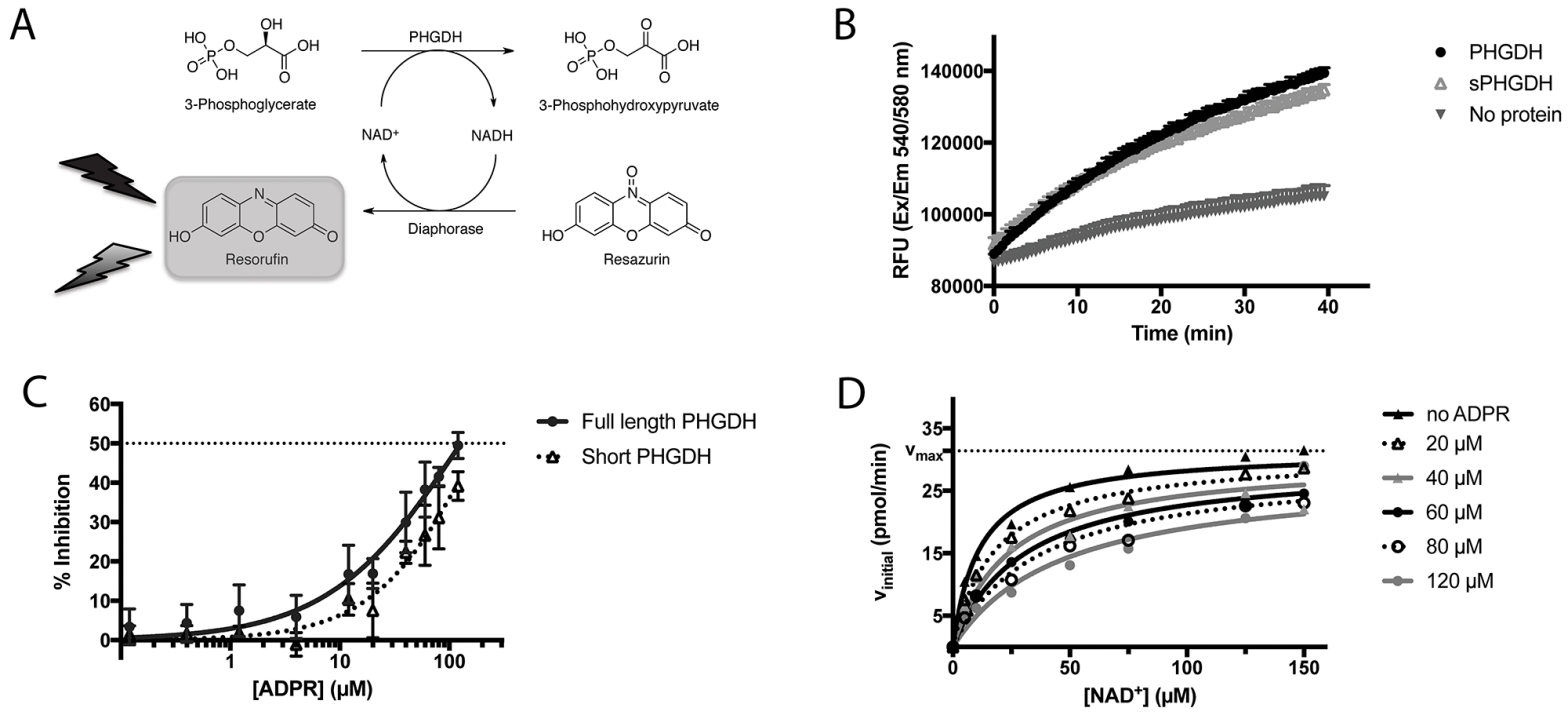
PHGDH and sPHGDH *in vitro* activity **(A)** Schematic representation of the biochemical PHGDH activity measurement. Enzymatic activity of human recombinant PHGDH or sPHGDH was measured in the direction of 3-PG oxidation to PHP by coupling the enzymatic reaction to the diaphorase-catalysed reduction of resazurin to the fluorescent product resorufin. The red fluorescent product, resorufin, was detected by monitoring emission at 580 nm upon excitation at 540 nm. **(B)** *In vitro* activity of PHGDH and sPHGDH. Enzymatic activity of PHGDH and sPHGDH was measured in the presence of 140 μM 3-PG and 25 μM NAD^+^ using 75 nM purified human PHGDH or sPHGDH. The propagation of the reaction was followed over time for 40 minutes after the addition of NAD^+^. Analysis was performed in GraphPad Prism by determining the initial velocity from the slope of increase in fluorescence over time and corrected for baseline increase in fluorescence in the absence of protein. **(C)** Inhibition of PHGDH/sPHGDH activity by ADPR. Enzymatic activity of PHGDH and sPHGDH was measured in the presence of increasing concentrations of ADPR (0.5 – 120 μM). Per cent inhibition was calculated by comparison to control samples containing no inhibitor (0 % inhibition) and a control without enzyme (100 % inhibition). Graph represents mean and standard deviation of three independent experiments with two intra-assay repeats. Data were analysed using nonlinear regression (log (inhibitor) *vs*. response – variable slope) in GraphPad Prism. **(D)** Mechanism of PHGDH inhibition by ADPR. *In vitro* activity of recombinant human PHGDH in the presence of varying concentrations of cofactor NAD^+^ (0 – 150 μM) and constant substrate concentration (3-PG, 140 μM), and varying amounts of ADPR (0 - 120 μM). Initial velocity was determined from the increase in fluorescence over time, and plotted against increasing concentrations of NAD^+^. The data were fitted to the mixed-model equation in GraphPad Prism. A representative graph of three independent experiments is shown with each condition assessed in duplicate. The initial velocity was determined as the mean of the three independent experiments.

In its natural environment, PHGDH catalyzes the oxidation of 3-PG to PHP, using NAD^+^ as oxidant. However, as PHGDH has a 400-fold higher affinity for NADH than NAD^+^, as determined by isothermal titration calorimetry (Figure 2), previously reported spectrophotometric PHGDH activity assays have mainly measured its activity in the direction of NADH oxidation [12, 13]. To perform the assay in this non-physiological direction, PHP is needed as substrate in addition to the cofactor NADH. Because PHP is no longer commercially available, it has become desirable to generate an assay that works in the physiological “forward” direction of NAD^+^ reduction. For the reaction to proceed in this direction, at least one product of the reaction (NADH or PHP) must be continuously removed from the reaction mixture. To characterize PHGDH activity in kinetic studies, we developed an assay in which the PHGDH-catalyzed reaction was coupled to a resazurin reduction reaction that uses NADH as cofactor, allowing for the continuous regeneration of NAD^+^ (Figure 3A). Using this assay, a K_m_ value of 186.7 ± 16.1 μM was determined for the oxidation of 3-PG (Supplementary Figure 1). Under the same reaction conditions, a truncated form of PHGDH (termed sPHGDH, residues 3-314, comprising the substrate and cofactor-binding domains) was shown to retain 75 % activity compared to PHGDH, despite lacking the regulatory ASB and ACT domains (Figure 3B).

### ADPR is an NAD^+^ competitive inhibitor of PHGDH

One possible approach to the targeting of PHGDH for anticancer drug discovery would be to develop a competitive inhibitor of cofactor binding. To confirm the feasibility of this approach, we have investigated the inhibitory mechanism of ADP-ribose (ADPR), a cofactor analogue that has been reported previously to be a competitive inhibitor of a different dehydrogenase, rabbit glyceraldehyde dehydrogenase [14].

In the *in vitro* enzyme activity assay, ADPR was able to inhibit full-length PHGDH activity by 50 % at the maximum tested dose of 120 μM, without affecting the coupled NADH recycling reaction (Figure 3C, Supplementary Figure 2). In addition, ADPR at a concentration of 120 μM also inhibited the activity of sPHGDH by about 40 %, showing that the effect of ADPR is independent of the regulatory domains of PHGDH, which together with the structural similarity of NAD^+^ and ADPR, suggested competitive inhibition. This hypothesis was confirmed by classical Michaelis-Menten kinetics of the PHGDH-catalyzed reaction in the presence of increasing amounts of APDR, showing similar V_max_ (31.4 ± 1.2 pmol min^-1^) and K_m_ (11.4 ± 2.1 μM) values (Figure 3D).

### Synergistic binding of cofactor, substrate and substrate analogues

**Figure 4:**
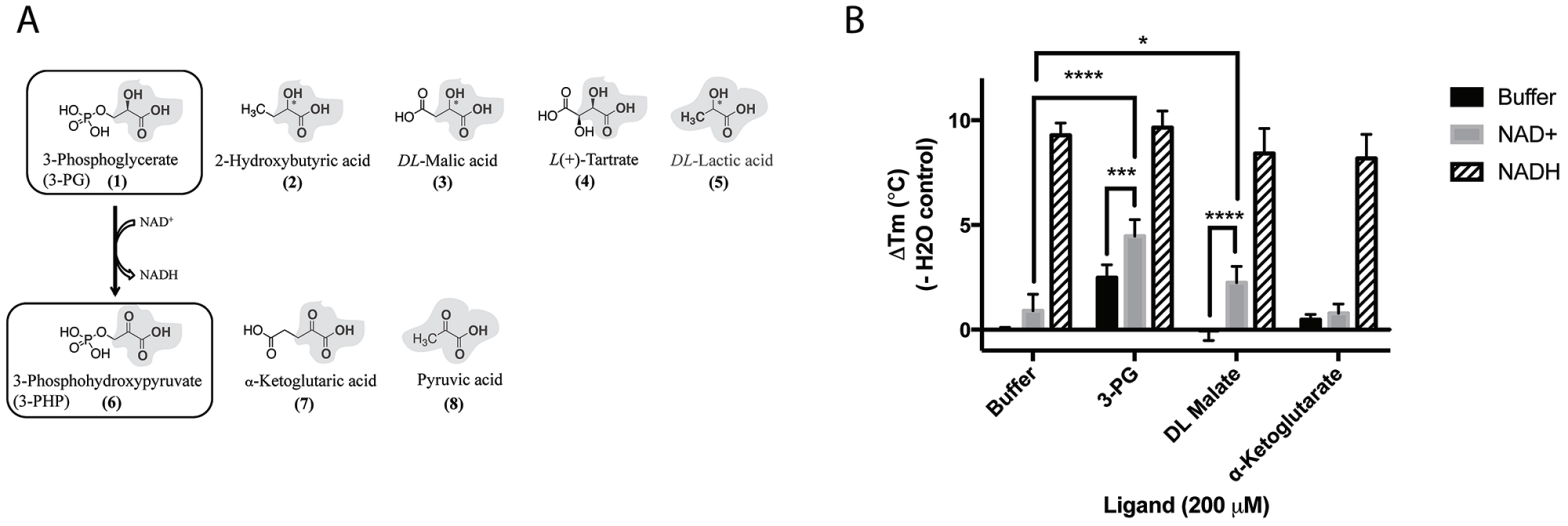
Structures and synergistic binding with NAD^+^ to PHGDH of the substrate 3-PG and the product 3-PHP and their analogues **(A)** For 3-phosphoglycerate (upper panel) as well as 3-phosphohydroxypyruvate (lower panel) and its analogues,the 2-hydroxypropanoic acid, or 2-oxopropanoic acid respectively, moieties are highlighted in bold. **(B)** Thermal denaturation of 1 μM PHGDH in the presence of 0.2 mM NAD^+^ or 0.2 mM NADH alone and in combination with 0.2 mM substrate analogues, was measured. Graphs represent mean and standard deviation of three independent experiments with two replicates per experiment. Statistical analysis was performed in GraphPad Prism (two way ANOVA). ^*^= p < 0.1, ^***^ = p < 0.005, ^****^ = p < 0.0005.

**Figure 5:**
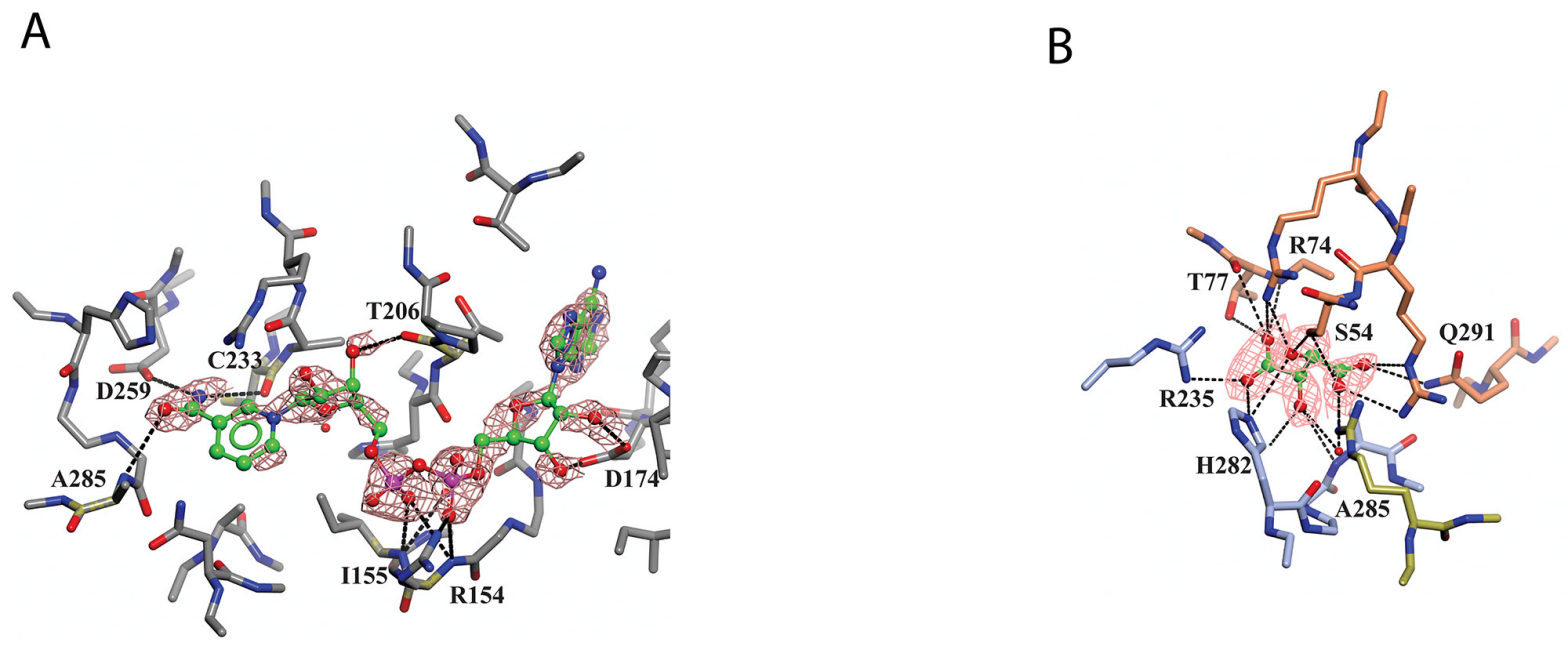
Binding of NAD^+^ and *L*-tartrate to sPHGDH Human sPHGDH structure showing interactions with cofactor NAD^+^ **(A)** and substrate analogue *L*-tartrate **(B)**. (A) sPHGDH and NAD^+^ are coloured by atom type and hydrogen bonds are indicated as dashed lines. (B) sPHGDH and *L*-tartrate are coloured by atom type with carbon atoms of sPHGDH coloured in coral (lid domain) and light blue (core domain) depending on which domain they belong to. Electron density map (2F_o_-F_c_ map) for NAD^+^ and *L*-tartrate is contoured at 1.3 electrons/A^3^ and is shown as orange mesh.

The binding of 3-PG (**1**), *DL*-malate (**3**) and other substrate and product analogues was investigated using differential scanning fluorimetry. The substrate analogues were similar in size to 3-PG and all contained a 2-hydroxypropanoic acid moiety (Figure 4A). Although PHP (**6**), the physiological binding partner of PHGDH, was not available commercially, two PHP analogues that share with PHP the 2-oxopropanoic acid moiety, namely α-ketoglutarate (**7**) and pyruvate (**8**), could be tested (Figure 5). Of the substrate and product analogues tested, only 3-PG (**1**) and α-ketoglutarate (**7**) induced a shift in the melting temperature of PHGDH greater than or equal to 0.5 °C, and only the natural substrate 3-PG (**1**) resulted in a statistically significant increase in T_m_ of PHGDH of 2.5 ± 0.6 °C (Figure 4B). Although *DL*-malate (**3**) was not seen to bind to PHGDH on its own in this assay, binding could be detected in the presence of NAD^+^, indicating a synergistic binding mechanism. The increase in T_m_ seen with 0.2 mM NAD^+^ alone was 0.9 ± 0.7 °C, whereas in combination with *DL*-malate (**3**) this value increased to 2.2 ± 0.7 °C (Supplementary Figure 4). A similar effect was seen upon combination of NAD^+^ and 3-PG (**1**) with a significant increase in melting temperature of 4.5 ± 0.7 °C. This synergistic stabilization effect seen upon co-incubation of NAD^+^ with the natural substrate 3-PG (**1**) or the analogue *DL*-malate (**3**) was unique to the oxidized cofactor and was not seen when combining NADH with substrate analogues. However, the increase in T_m_ seen in the presence of 0.2 mM NADH alone (9.3 ± 0.5 °C) was much more prominent, potentially masking stabilization effects due to substrate/ product (analogue) binding (Figure 4B).

### Co-crystal structure of sPHGDH with NAD^+^ and *L*-tartrate reveals a domain movement upon substrate binding

Although co-crystallization of sPHGDH with 3-PG and different substrate analogues was attempted, sPHGDH only co-crystallized with NAD^+^ and *L*-tartrate (Table 1, PDB 5N6C). In agreement with the deposited structure of sPHGDH, two molecules were present in the crystallographic asymmetric unit (ASU). However, while both molecules had NAD^+^ bound to the cofactor sites, *L*-tartrate was only present in one molecule, allowing comparison of substrate-bound and unbound states. In both domains, NAD^+^ was bound in the same way, forming various hydrogen bonds with the enzyme (Figure 5A). The nicotinamide moiety interacted with the backbone of the protein (A285 and C233), but also with the side chain of D259. The hydroxyl-groups of the sugar moieties were also involved in hydrogen bonds with the protein backbone (T206) and side chain of D174. The phosphate linker interacted with the main chain of R154 and I155 and also the side chain of R154 (Figure 5A).

**Table 1:** Diffraction data collection and refinement statistics (molecular replacement)

	sPHGDH – NAD^+^ and *L*-tartrate (PDB ID: 5N6C)
***Data collection***	
Beamline	Diamond Light Source, I04-1
Wavelength (Å)	0.92
Space group	P 2_1_
Cell dimensions	
*a, b, c* (Å)	*43.5, 109.7, 66.9*
α, β, γ (°)	90.0, 94.0, 90.0
No. unique reflections	29527 (2224)
Resolution (Å)	57.0 - 2.2 (2.3 – 2.2)
R_merge_	0.06 (0.59)
I/σI	12.6 (2.3)
Completeness (%)	98.0 (98.8)
Redundancy	3.8
***Refinement***	
Ligand	NAD^+^ and *L*-tartrate
Resolution (Å)	66.7-2.3
No. reflections (all/free)	27427 / 1319
Rwork / Rfree	0.21 / 0.25
Average B factor (Å^2^)	
All atoms	26
Protein	8
Water	36
R.m.s. deviations	
Bond lengths (Å)	0.02
Bond angles (°)	2.21
***Molprobity***	
Ramachandran favoured (%)	96.0

Values in parentheses are for highest-resolution shell. R.m.s., root-mean-square.

**Figure 6:**
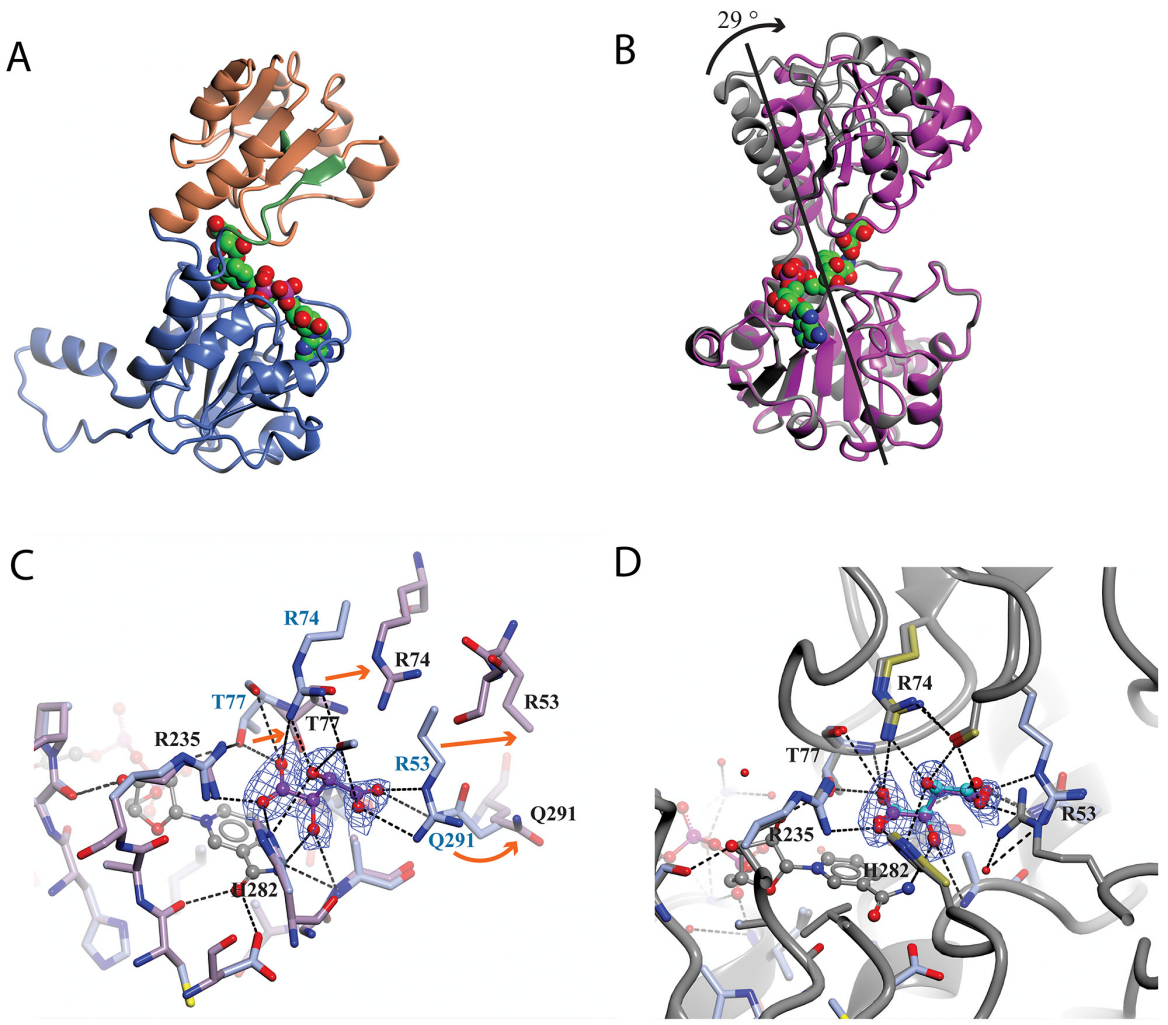
Analysis of the domain movement in a co-crystal structure of sPHGDH with *L*-tartrate and NAD^+^ The protein crystal structure was analysed using the DynDom web server. **(A)** Structure of sPHGDH chain A with different domains highlighted: fixed domain in blue, mobile domain in orange, hinge domain in green. The cofactor NAD^+^ is shown as spheres coloured by atom type. **(B)** Superposition of the two molecules in the ASU. Structures containing *L*-tartrate and without *L*-tartrate are coloured in purple and grey, respectively. NAD^+^ and *L*-tartrate are coloured by atom type and displayed as spheres. **(C)** Changes in the substrate-binding site of sPHGDH observed upon binding of *L*-tartrate. Chain A (light blue) with *L*-tartrate (purple) and NAD^+^ (grey) bound to the active site superposed onto chain B (lilac) with only NAD^+^ bound to the active site. Movement of individual amino acids involved in *L*-tartrate binding indicated with orange arrows. **(D)** Chain A (light blue/gold) with *L*-tartrate bound (purple) superposed onto chain A (grey) of sPHGDH with NAD^+^ and *D*-malate bound (cyan) (PDB 2G76). New hydrogen bonds are formed between the amino acids highlighted in gold and the additional OH-group of *L*-tartrate compared to binding of *D*-malate. Electron density map (2F_0_-F_c_ map) for *L*-tartrate is contoured at 1.3 electrons/A^3^ and is shown as blue mesh.

The substrate analogue, *L*-tartrate, interacted with both the lid and the core domain of sPHGDH, through a network of hydrogen bonds (Figure 5B). *L*-Tartrate formed hydrogen bonds to R235, H282 and A285 of the core domain and to R53, S54, R74, T77 and Q291 of the lid domain. In the absence of *L*-tartrate, the lid domain, which otherwise closed over the substrate-binding site, underwent a rigid domain movement. In order to characterize the movement, the two molecules in the ASU were compared using the DynDom webserver [149, 150]. This analysis suggested three structural sub domains: a fixed core domain (aa 100-287), a mobile lid domain (aa 9-93 and aa 289-301) and a hinge domain (aa 94-99 and aa 302) (Figure 6A). Interestingly, superposition of the two conformations with and without bound *L*-tartrate showed that the lid domain had rotated by 29° upon substrate binding (Figure 6B). Upon closer inspection of the substrate-binding sites in both chains, distinct movements of the amino acids involved in *L*-tartrate binding were evident. In the presence of *L*-tartrate, a subset of amino acids either moved into the binding site (Arg74, Arg53), so that Arg53 interacted with *O* and *O*^1^ of *L*-tartrate and Arg74 with *O*^2^ and *O*^5^ of the substrate analogue, or adopted alternate rotamers that oriented their side-chains towards the binding site (Gln291) (Figure 6C and 6D).

### NAD^+^ analogues can substitute for NAD^+^ in the catalytic reaction of PHGDH

NAD^+^ analogues were used to study the effect of changes around the nicotinamide-binding subsite on the enzymatic activity and stability of PHGDH and to understand what functional groups would be tolerated in this part of the cofactor-binding site.

**Figure 7:**
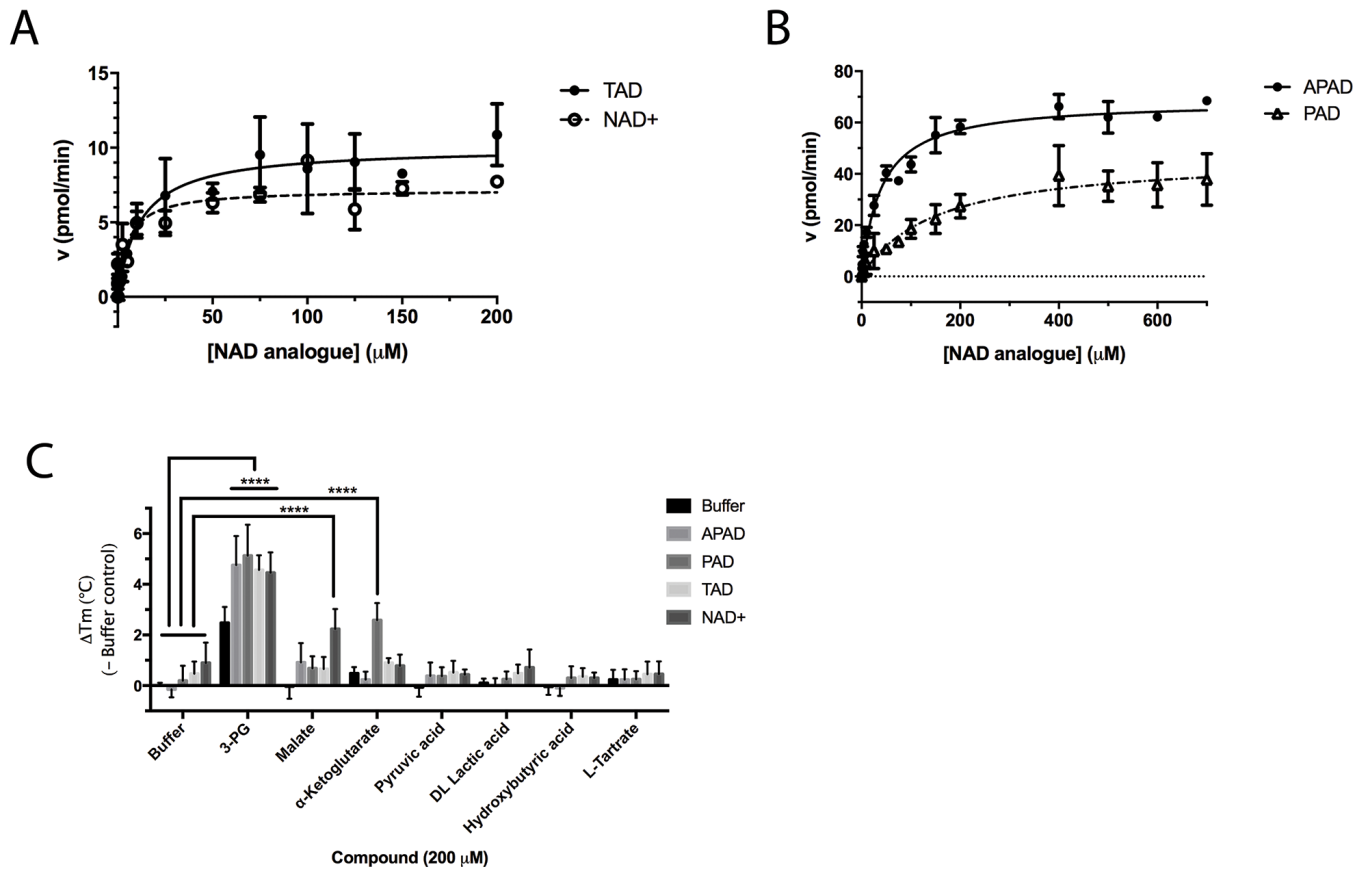
Enzyme kinetic data for PHGDH with various substrate analogues *In vitro* PHGDH activity assay containing recombinant human PHGDH and increasing concentrations of NAD^+^ or TAD **(A)** or the NAD^+^ analogues PAD or APAD **(B)**. The increase in fluorescence due to conversion of NAD^+^ to NADH was measured and initial velocity determined by linear regression. The initial velocity was plotted against cofactor/ cofactor analogue concentration and data fitted to a Michaelis-Menten model. All curve fitting was performed using GraphPad Prism. Data plotted represents mean and standard deviation of two independent experiments with two replicates per experiment. **(C)** Change in T_m_ (ΔT_m_) of PHGDH upon addition of NAD^+^ analogues in combination with substrate/ substrate analogues. Thermal denaturation of 1 μM PHGDH in the presence of 0.2 mM NAD^+^ analogue in combination with 0.2 mM substrate/ product analogue. Graphs represent mean and standard deviation of at least two independent experiments with two replicates per experiment. Statistical analysis was performed in GraphPad Prism (two way ANOVA). ^****^ = p < 0.0001.

Thionicotinamide (TAD), acetylpyridine (APAD) and Thionicotinamide (TAD), acetylpyridine (APAD) and pyridinealdehyde adenine dinucleotide (PAD) were tested in the enzyme activity assay in place of NAD^+^ and all were found to allow the enzymatic reaction to proceed while not affecting the enzymatic activity of the recycling enzyme (Supplementary Figure 3). In particular, TAD was found to be very similar to NAD^+^ in terms of maximum velocity (V_max_) (10.0 and 7.2 pmol min^-1^ for TAD and NAD^+^ respectively) and k_cat_ (0.45 and 0.32 min^-1^ for TAD and NAD^+^ respectively). In contrast, V_max_ for APAD and PAD was higher than NAD^+^. APAD showed a 10-fold increase in k_cat_ compared to NAD^+^, whereas PAD showed a slightly reduced k_cat_ (Figure 7A and 7B, Table 2).

**Table 2:** Summary of calculated parameters of PHGDH enzyme activity. ^a^R is used to denote the ADP-ribose moiety. ^b^ ΔT_m_ of PHGDH in combination with 3-PG (200 μM) and cofactor (analogue) (200 μM) compared to buffer only control. 3-PG (200 μM) alone results in an increase in T_m_ of PHGDH of 2.5 ± 0.6 °C.

	Structure of nicotinamide moiety^a^	V_max_ (pmol/min)	k_cat_ (min-1)	K_m_ (μM)	ΔT_m_^b^ (-Buffer control) (°C)
**NAD**^+^	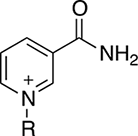	7.2 ± 0.4	0.3 ± 0.0	5.3 ± 1.6	4.5 ± 0.8
**TAD**	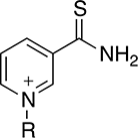	10.0 ± 0.6	0.45 ± 0.0	11.8 ± 3.5	4.6 ± 0.6
**APAD**	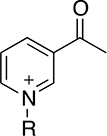	68.4 ± 1.9	3.04 ± 0.1	39.4 ± 4.9	4.8 ± 1.1
**PAD**	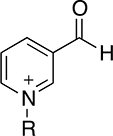	46.8 ± 3.8	0.21 ± 0.2	147.7 ± 34.9	5.1 ± 1.2

All cofactor analogues tested could replace NAD^+^ and sustain PHGDH activity. In order to compare their binding affinities with NAD^+^, a DSF assay was performed (Figure 7C). All the cofactor analogues on their own stabilized PHGDH less than NAD^+^, with APAD showing no increase in T_m_, suggestive of relatively low affinity of PHGDH for APAD in the absence of 3-PG (**1**). This is concordant with the weaker affinity of PHGDH for APAD giving a higher K_m_ compared to the natural cofactor NAD^+^ in the *in vitro* enzyme activity assay. In combination with 3-PG, all analogues showed the same synergistic behavior, increasing the T_m_ of PHGDH to the same extent as NAD^+^. This result is even more striking considering that the NAD^+^ analogues yielded only small increases in T_m_ of PHGDH when tested in the absence of substrate analogues. This indicates that the binding of 3-PG might induce a conformational change of the protein that increases accessibility to the cofactor binding pocket and permits binding of cofactor analogues or slows down their dissociation. Intriguingly, for NAD^+^ analogues in combination with other substrate analogues, a significant change in T_m_ was only seen for PAD together with α-KG (ΔT_m_ = 2.6 ± 0.7 °C) (Figure 7C, Supplementary Figure 4).

## DISCUSSION

A crystal structure of a truncated form of human PHGDH comprising the nucleotide- and substrate-binding regions revealed *D*-malate, a substrate analogue, bound to the substrate-binding pocket. Investigation of the binding of malate and other substrate analogues of 3-PG and PHP in a DSF assay highlighted *L*-tartrate and α-KG as weak stabilizers of PHGDH, whereas the natural substrate 3-PG gave a substantial increase in T_m_.

*E. coli* PHGDH can utilize α-KG as a substrate, although human as well as rat and *M. tuberculosis* PHGDH have been reported not to share this ability [8]. Nevertheless, a recent study found human PHGDH to be able to catalyze the NADH-dependent reduction of α-KG to *D*-2-hydroxyglutarate, an oncometabolite associated with brain cancer and acute myeloid leukemia [11]. In this context our work shows for the first time the biochemical basis for this finding, by confirming the binding of α-KG to human PHGDH, consistent with the suggestion that PHGDH could effect this cancer-linked transformation *in vivo*. This result confirms that human PHGDH displays a degree of substrate promiscuity and can bind alternative substrates to the primary substrate 3-PG. This promiscuity could be of special relevance in determining the role of PHGDH in certain cancer forms that depend on PHGDH activity (often associated with elevated PHGDH expression levels) beyond its role in serine synthesis. In such cancer cell lines PHGDH depletion resulted in decreased proliferation effects, which could not be rescued by increased serine supplementation to the media [2, 4].

Binding of substrate analogues was also investigated in combination with the cofactor NAD^+^ as possible interdependence of substrate and cofactor binding is suggested by the close proximity of their respective binding sites. Indeed, the combination of 3-PG and NAD^+^ increased the T_m_ significantly, and by more than the simple addition of the effects seen with the two compounds separately. The same was found to be true for *DL*-malate where the presence of NAD^+^ appeared to be necessary for the substrate analogue to bind at all, although NAD^+^ was able to bind on its own. Thus for *DL*-malate and NAD^+^, cofactor binding seems to be a necessary event to permit binding of the substrate analogue, whereas for 3-PG a different obligatory binding order is suggested based on the DSF data, since 3-PG alone is able to provide a substantial T_m_ shift. Interpretation of these results might be complicated by the observation that PHGDH appears to co-purify with NAD^+^ or NADH, as suggested by the relatively high OD_260_:OD_280_ ratio of 0.9 for untreated preparations of PHGDH compared to a reported ratio of OD_260:_OD_280_ of 0.57 for pure protein [15]. Taking into account that a certain amount of cofactor is already bound to the purified protein when we performed the experiments, it seems likely that the order of binding must be cofactor before substrate. To support this preferred binding order, our co-crystal structure of sPHGDH with *L*-tartrate, revealed one subunit containing L-tartrate and NAD^+^, and one subunit containing only NAD^+^. Comparison of the two molecules showed that in the presence of *L*-tartrate a movement of the lid domain towards a more closed substrate pocket had occurred, consistent with the hypothesis that the substrate binds after the cofactor. For PHGDH from *M. tuberculosis*, another type I PHGDH, the substrate is reported to bind before the cofactor, although this catalytic mechanism was investigated in the (non-physiological) direction of PHP reduction [8].

An understanding of the promiscuity of the substrate and co-factor binding sites in PHGDH is critical to define its role, not only in healthy and disease models, but also for the development of PHGDH inhibitors targeting the active site. Using DSF, we have shown that several substrate or product analogues are able to bind to the substrate-binding site. However, we also used NAD^+^ analogues, such as APAD, TAD and PAD to investigate if they would be able to interact with PHGDH. These analogues are known to be able to replace NAD^+^ in reactions catalyzed by a variety of enzymes [16, 17] and this was also found to be true for PHGDH. In particular, APAD showed a ten-fold higher apparent k_cat_ than NAD^+^, which is likely due to its higher oxidation potential, which in turn may support a higher rate in the redox reaction [18]. Interestingly, analysis of the binding affinities of the cofactor analogues by DSF showed that none of the analogues stabilized PHGDH to the same extent as NAD^+^, with *e.g.* no stabilization seen with APAD. This is consistent with an elevated K_m_ (*i.e.* lower affinity for the substrate) for APAD as a cofactor analogue in the sPHGDH-catalyzed reaction. If release of the product of APAD reduction is rate limiting, this lower affinity might also contribute to a higher apparent k_cat_ for APAD relative to NAD^+^. While cooperative stabilization of PHGDH by most substrates was dependent on the identity of the cofactor analogue used, all cofactor analogues were able to exert a stabilizing effect in combination with 3-PG.

With the exception of α-KG in combination with PAD, a cooperative stabilization of PHGDH was not observed with other substrate analogue/co-factor analogue combinations. A significant increase in the T_m_ of PHGDH in the presence of PAD and α-KG, but not upon combination of α-KG with other cofactor analogues, is unlikely to be due to a difference in the electron distribution around the aromatic ring: the aldehyde function of PAD, like the amide substituent of NAD^+^ has an electron withdrawing inductive and resonance effect on the aromatic ring. Therefore it is more likely due to the difference in the size of the moiety, with hydrogen replacing the NH_2_ group in PAD. This would also explain why the effect is only seen with PAD and not with APAD and TAD, with the latter two having a methyl group or an NH_2_ group at the position of the hydrogen, respectively.

In summary, we have investigated the substrate binding of PHGDH by solving the crystal structure of sPHGDH with and without the substrate analogue *L*-tartrate bound to the catalytic site, revealing a distinct domain movement upon substrate binding. We have shown that different substrate analogues are tolerated in the protein’s binding site, and that substrate binding can increase the thermal stability of PHGDH. The cofactor-binding site also tolerates the binding of NAD^+^ analogues with modifications around the nicotinamide moiety, all of which are able to both bind and sustain enzymatic activity. These findings support the hypothesis that PHGDH may be involved in more enzymatic reactions than its primary role in 3-PG oxidation and we suggest that such additional roles could be especially relevant to explain the role of PHGDH in cancer. The substrate and cofactor promiscuity we have seen with PHGDH suggests that substrate or co-factor competitive inhibitors may well be tolerated in the PHGDH active site, opening up new approaches for the development of PHGDH inhibitors. Exploring this strategy may be timely, since other approaches to PHGDH inhibition have already been shown to reduce cancer progression in mice.

## MATERIALS AND METHODS

### Preparation of recombinant human PHGDH and sPHGDH

pNIC28-Bsa4 plasmids containing the human PHGDH cDNA encoding the full-length enzyme (aa 1-533) as well as a truncated version (sPHGDH, aa 3-314) were kindly donated by Wyatt Yue (SGC Oxford, Oxford, UK). Both plasmids are fused with an N-terminal His_6_-tag and a Tobacco Etch Virus (TEV) protease recognition site. The proteins were expressed in *E. coli* Rosetta (DE3) grown in TB medium (Sigma Aldrich, St. Louis, MO, USA) containing 50 μg/mL kanamycin and the purification protocol adapted from SGC Oxford (http://www.thesgc.org/structures/2g76#mand). The bacterial cells were grown at 37 °C to an optical density at 600 nm of 0.8-0.9. PHGDH/ sPHGDH expression was induced by addition of isopropyl β-D-1-thiogalactopyranoside (IPTG) to a final concentration of 0.5 mM. Cells were grown for 18 hours at 25 °C before harvesting by centrifugation and subsequent resuspension in 50 mM NaH_2_PO_4_, pH 8, 10 mM imidazole, 300 mM NaCl, 0.5 mM TCEP, 0.01 mg/mL DNase, 0.05 mg/mL RNase, 0.25 mg/mL lysozyme, 5 mM MgCl_2_ and protease inhibitor. The cell suspension was sonicated on ice, followed by centrifugation. The lysate was applied to a HisTrap Ni-Sepharose column and the proteins eluted with an imidazole gradient to a maximum of 250 mM imidazole. The His_6_-tag was cleaved by incubation with TEV protease at a mass ratio of 1:25 (protease: protein) at 4 °C for 16 hours. The mixture was applied to a gravity flow column containing Ni-Sepharose beads to capture TEV protease and His_6_-fragments. The protein was further purified by size exclusion chromatography on a Superdex™ 75 26/60 column (GE Healthcare) in 25 mM HEPES, pH 7.5, 100 mM NaCl, 0.5 mM TCEP. The protein containing fractions were combined, concentrated and buffer exchanged into low salt buffer (20 mM Tris, pH 8, 40 mM NaCl, 0.5 mM TCEP) using a PD-10 column (GE Healthcare, Little Chalfont, UK). The protein was further purified by anion exchange chromatography using a linear gradient up to 1 M NaCl (high salt buffer).

### Enzyme activity assay

The enzymatic activity of PHGDH or its catalytic domain (sPHGDH) was measured in the direction of 3-PG oxidation to PHP by coupling the reaction with a resazurin reduction reaction to allow fluorescence detection [19]. The assay mixture contained 18 mM BIS TRIS propane, pH 7.5, 1.7 mM EDTA, pH 7.5, 10 μM Triton X-100, 3.3 mM glutathione, 140 μM 3-PG (Santa Cruz Biotechnology, Dallas, Texas, USA), 25 μM NAD^+^ and 75 nM purified human PHGDH or sPHGDH. The assay was performed in black 96-well plates (Sterilin Ltd, Gwent, UK) in 150 μL total volume of which 25 μL was the NAD^+^ recycling mixture containing the diaphorase and resazurin (Amplite Fluorimetric assay buffer, Stratech Scientific Ltd., Suffolk, UK). The fluorescence was measured on an Omega Platereader (BMG Labtech, Ortenberg, Germany) over 40 – 50 minutes. Analysis was performed in GraphPad Prism by determining the initial velocity from the slope of increase in fluorescence over time. The initial velocity was corrected for baseline increase in fluorescence in the absence of protein.

### Isothermal titration calorimetry

ITC experiments were performed in 25 mM HEPES, pH 7.5, 100 mM NaCl, 0.5 mM TCEP at 25 °C in a MicroCal iTC200 instrument (GE Healthcare). To remove residual bound cofactor, sPHGDH was incubated with 0.5 equivalents of activated charcoal for 30 minutes at room temperature prior to performing the ITC experiments. The charcoal was removed by centrifugation at 13 000 x g, 4 °C for 10 minutes, followed by filtering the supernatant through a PD10 column. 1 x 0.5 μL, followed by 17 x 2 μL injections of ligands into protein with 120 seconds spacing between the injections was conducted. The thermodynamic parameters of the reactions were determined following peak integration and best-fit of data to the one-site binding model using ORIGIN version 7.0 (OriginLab).

### Differential scanning fluorimetry

The assay mixture contained 25 mM HEPES, pH 7.5, 100 mM NaCl, 0.5 mM TCEP, 1 x Sypro Orange (Thermo Fisher Scientific, Waltham, MA, USA) and 1 μM protein. Ligand solutions were prepared in 25 mM HEPES, pH 7.5, and added at 200 μM to the assay. The heat denaturation curves were recorded using an RT-PCR instrument (ViiA7, Applied Biosystems, Warrington, UK). The temperature was increased by 3 °C/min starting at 25 °C up to 95 °C and the fluorescence of Sypro Orange measured with excitation and emission wavelengths of 470 and 570 nm, respectively. Analysis was performed using the Boltzmann equation in GraphPad Prism.

### Crystallization, X-ray crystallographic data collection, structure determination and analysis

For co-crystallization, sPHGDH at 15 mg/mL (in 25 mM HEPES, pH 7.5, 100 mM NaCl, 0.5 mM TCEP) was mixed with substrate analogue and incubated at 4 °C for 16 hours. The mixture was screened for crystallization against sparse matrix screens: Index (Hampton Research, Aliso Viejo, CA, USA), JCSG^+^, Structure, Proplex and Morpheus (all from Molecular Dimensions, Newmarket, Suffolk, UK) in 96-well MRC crystallization plates (Molecular Dimensions, Newmarket, Suffolk, UK) by the sitting drop vapour diffusion method. Plates were set up using a Mosquito LCP liquid handler (TTP Labtech, Melbourn, UK) with two drops of 300 nL protein (15 mg/mL) mixed with 300 or 600 nL precipitant and a shared reservoir solution of 70 μL precipitant. Crystal trials were stored at 4 or 20 °C. Crystals thus obtained were cryo-protected in reservoir solution (0.1 M Bis TRIS buffer, pH 6.5, 0.3 M NH_4_Ac, 17 % PEG3350) supplemented with ligand and 20 % (w/v) PEG400 before being flash cooled in liquid nitrogen. Diffraction data were collected on beamline I04, Diamond Light Source. Data were processed using xia2, an automated combination of XDS [20], XSCALE [20] and programs from the CCP4 suite [21] and used for molecular replacement with PHASER [22], run through the CCP4i2 GUI, using the published structure of human PHGDH (PDB 2G76) as the search model. The structure was refined using iterative cycles of manual model correction in COOT [23] followed by refinement with Refmac5 [24] within CCP4 [21]. Models were validated using the validation tools available in COOT, and MolProbity [25]. Figures were produced using CCP4mg [26].

## SUPPLEMENTARY MATERIALS FIGURES



## Abbreviations

PHGDH: 3-phosphoglycerate dehydrogenase
3-PG: 3-phosphoglycerate
PHP: phosphohydroxypyruvate
ADPR: ADP-ribose
TAD: thionicotinamide adenine dinucleotide
APAD: acetylpyridine adenine dinucleotide
PAD: pyridinealdehyde adenine dinucleotide
DSF: differential scanning fluorimetry
α-KG: α-ketoglutarate
T_m_: melting temperature
ITC: isothermal titration calorimetry
TB: Terrific broth
IPTG: isopropyl β-D-1-thiogalactopyranoside
TCEP: tris (2-carboxyethyl) phosphine
