# Supplementary material for: Structural insights into the enzymatic activity and potential substrate promiscuity of human 3-phosphoglycerate dehydrogenase (PHGDH)
Source: Oncotarget. 2017 Nov 6;8(61):104478–91. doi: 10.18632/oncotarget.22327 (PMC5732821; doi:10.18632/oncotarget.22327)
Supplement: Supplementary file 1 [file oncotarget-08-104478-s001.pdf]

Structural insights into the enzymatic activity and potential substrate promiscuity of human 3-phosphoglycerate dehydrogenase (PHGDH)

SUPPLEMENTARY MATERIALS

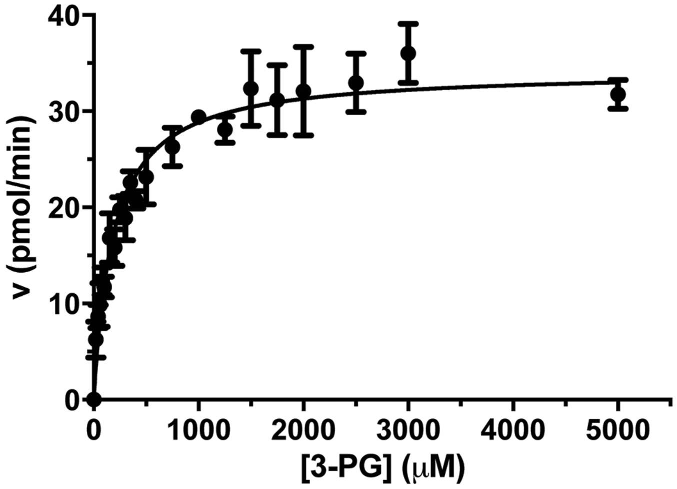

Supplementary Figure 1: Determination of K_m values for PHGDH. Enzymatic activity of PHGDH was measured in the presence of increasing concentrations of 3-PG (0-5 mM) and 25 μM NAD^+ using 75 nM purified human enzyme. The propagation of the reaction was followed over time for 40 minutes after the addition of NAD+. The initial velocity from the slope of increase in fluorescence over time was determined and corrected for baseline increase in fluorescence in the absence of 3-PG. Determination of initial velocity by linear regression and Michaelis Menten analysis was performed in GraphPad Prism.

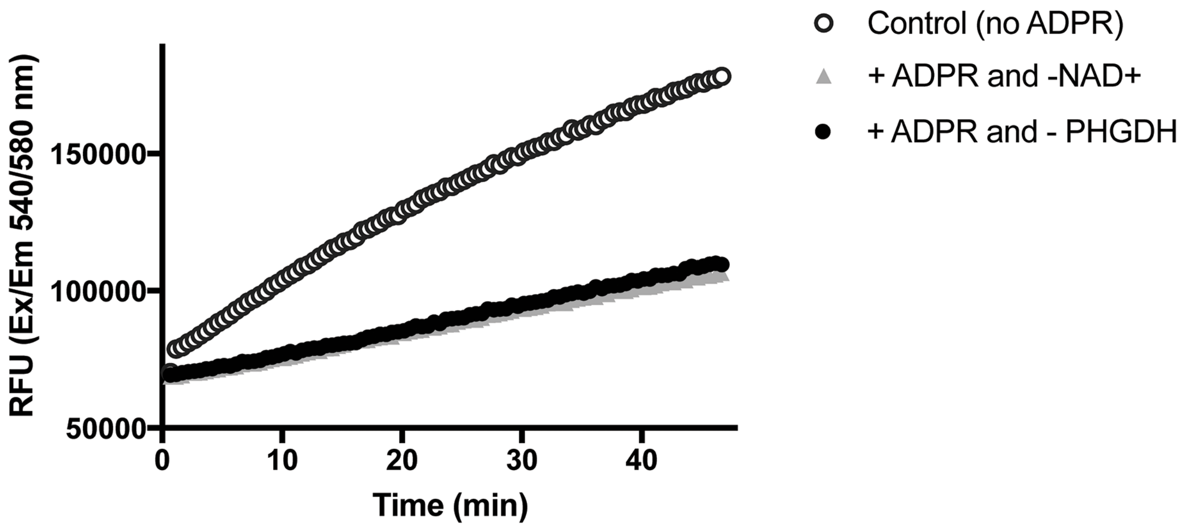

Supplementary Figure 2: ADPR does not affect recycling enzyme. Enzymatic activity assay was performed with and without PHGDH and ADPR resulting in a linear increase in RFU signal. There is no enzymatic activity in the samples lacking PHGDH or NAD^+, despite the presence of APDR.

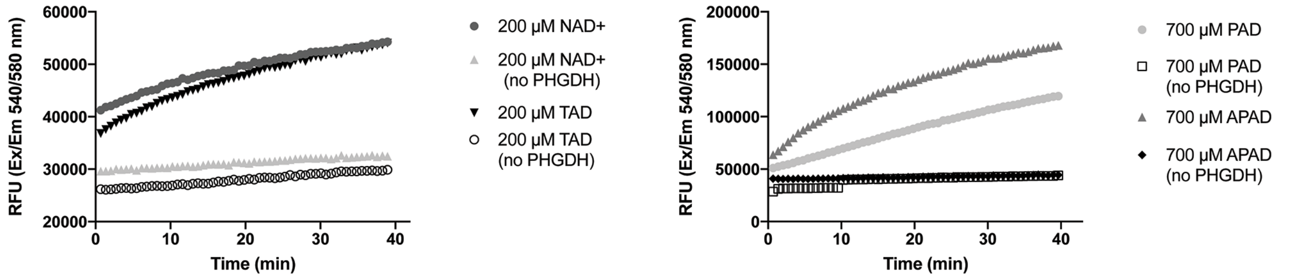

Supplementary Figure 3: Effect of NAD$^+$ analogues on recycling enzyme. Enzymatic *in vitro* measurements were performed with NAD$^+$ analogues at the highest concentration used in the assay in the presence and absence of PHGDH. The NAD$^+$ analogues do not serve as substrates/ cofactor for the recycling enzyme.

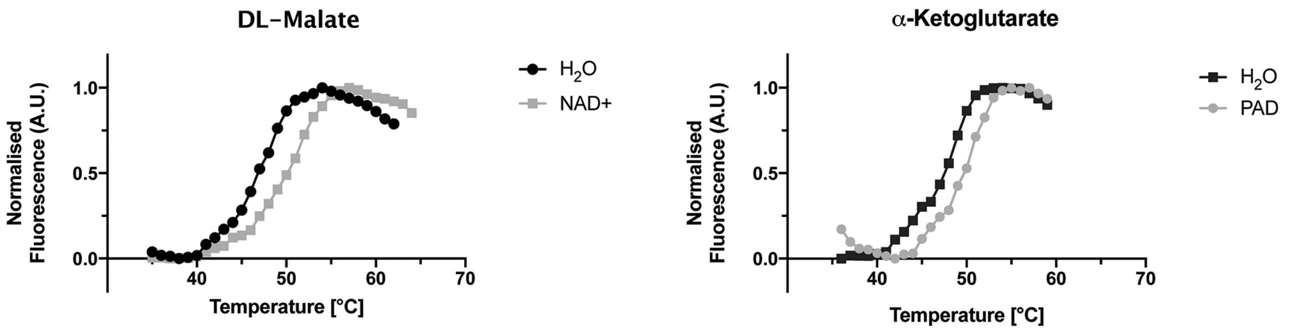

Supplementary Figure 4: Thermal denaturation curves of PHGDH in combination with NAD+ and DL-malate (left) or PAD and α-KG (right). Unfolding transition of 1 μM PHGDH with increasing temperature with and without 0.2 mM NAD+ or PAD in combination with 0.2 mM substrate analogue and in the presence of Sypro Orange.
